# Discovery and characterization of novel *Aspergillus fumigatus* mycoviruses

**DOI:** 10.1371/journal.pone.0200511

**Published:** 2018-07-25

**Authors:** Jan Zoll, Paul E. Verweij, Willem J. G. Melchers

**Affiliations:** Department of Medical Microbiology, Radboud University Medical Center, and Center of Expertise in Mycology Radboudumc/CWZ, Nijmegen, The Netherlands; Oklahoma State University, UNITED STATES

## Abstract

In the last few years, increasing numbers of viruses infecting fungi have been identified. In this study, we used an *in silico* approach for the analysis of deep RNA sequencing data in order to discover and characterize putative genomic ssRNA or dsRNA mycovirus sequences in *Aspergillus fumigatus*. RNA sequencing reads of *A*. *fumigatus* strains were mapped against the *A*. *fumigatus* Af293 reference genome. Unmapped reads were collected for *de novo* assembly. Contigs were analyzed by Blastx comparison with a mycovirus protein database. Assembled viral genomes were used as template for remapping of RNA sequencing reads. In total, deep RNA sequencing results from 11 *A*. *fumigatus* strains were analyzed for the presence of mycoviral genomic RNAs. In 9 out of 11 strains, putative mycoviral RNA genomes were identified. Three strains were infected with two different mycovirus species. Two strains were infected with Aspergillus fumigatus polymycovirus type-1 (AfuPmV-1). Four strains contained fully recovered genomic RNA of unknown narna-like viruses designated as Aspergillus fumigatus narnavirus-1 and Aspergillus fumigatus narnavirus-2 (AfuNV-1 and AfuNV-2). Both viruses showed 38% amino acid sequence identity to Beihai narna-like virus-21. Three strains contained partially recovered genomic RNA of an unknown narna-like virus. Two strains contained fully recovered genomic RNAs of an unknown partitivirus designated as Aspergillus fumigatus partitivirus-2 (AfuPV-2) which showed 50% amino acid sequence identity to Alternaria alternata partitivirus-1. Finally, one strain contained fully recovered genomic RNA of an unknown mitovirus designated as Aspergillus fumigatus mitovirus-1 (AfuMV-1) which showed 34% amino acid sequence identity to Sclerotina sclerotiorum mitovirus. *In silico* analysis of deep RNA sequencing results showed that a majority of the *A*. *fumigatus* strains used here were infected with mycoviruses. Four novel *A*. *fumigatus* RNA mycoviruses could be identified: two different Aspergillus fumigatus narna-like viruses, one Aspergillus fumigatus partitivirus, and one Aspergillus fumigatus mitovirus.

## Introduction

Recent studies have shown that mycoviruses are widespread among all major fungal taxa, including *Aspergillus* [1–4]. In the last decades, deep sequencing techniques revealed increasing numbers of newly discovered mycovirus species [2, 5, 6]. Based on genetic characteristics, several mycovirus genera can de defined. All known mycoviruses found in aspergilli were recently reviewed by Kotta-Loizou and Coutts [7]. The majority of the mycoviruses known so far possess double-stranded RNA (dsRNA) or single-stranded (ssRNA) genomes of either positive or negative sense. Most dsRNA mycoviruses have segmented genomes. Until now, only one single-stranded DNA (ssDNA) mycovirus with a circular genome has been described [8]. Double-stranded DNA (dsDNA) mycoviruses have not been discovered yet.

Little is known about the transmission of mycoviruses. Especially mycoviruses with RNA genomes are generally considered as endogenous viruses without an extracellular stage during virus replication. However, it was reported that Gemycircularvirus, a circular ssDNA virus, is transmitted via infectious particles [9]. Although several dsRNA and ssRNA mycovirus species encode for capsid protein, there is no evidence for the production of infectious viral particles in infected fungi. Spreading of mycoviruses is thought to occur primarily through anastomosis and infected conidia [7, 10].

Mycoviruses are usually detected by isolation of genomic dsRNA or a dsRNA replicative intermediate. Mycovirus RNAs are further characterized by purification from agarose gels and sequencing of cDNAs generated from de dsRNA fragments [11]. Deep sequencing is a more efficient approach to discover and indentify unknown viruses. In this study, we used the results of several transcriptomics experiments in *Aspergillus fumigatus*. In 9 out of 11 strains tested in this study, we found RNAs from six different mycoviral species. In this paper, we describe the molecular discovery and characterization of four novel *A*. *fumigatus* mycoviruses.

## Materials and methods

### Strains and RNA extraction

The search for mycovirus sequences made use of sequencing reads generated during transcriptome analysis of a series of *A*. *fumigatus* strains (Table 1). Strains were cultivated in Sabouraud dextrose agar slants (dextrose 40 g/L, peptone 10 g/L, agar 20 g/L, pH 5.6) for 5 days at 30°C. The conidia were harvested with wet cotton swabs and placed in Milli-Q containing 0.01% Tween 20. For inoculation, conidia were diluted in RPMI medium to a final concentration of 1x10^8^ conidia/mL.

**Table 1:** Mycovirus genomic RNA segments found in *Aspergillus fumigatus* strains.

Strain	Isolate	Cyp51A mutations	Azole resistance	Mycovirus genomic RNA	Number of genomic RNA segments
1	V130-14	none	susceptible	Aspergillus fumigatus narnavirus 3 (AfuNV-3)	1 (partial)
2	V145-13	none	susceptible	Aspergillus fumigatus partitivirus 2 (AfuPV-2)	2
				Aspergillus fumigatus narnavirus 3 (AfuNV-3)	1 (partial)
3	V145-33	TR46/Y121F/T289A	resistant	Aspergillus fumigatus narnavirus 2 (AfuNV-2)	1
				Aspergillus fumigatus mitovirus 1 (AfuMV-1)	1
4	V145-34	none	susceptible	Aspergillus fumigatus polymycovirus 1 (AfuPmV-1)	4
5	V145-40	none	resistant	Aspergillus fumigatus partitivirus 2 (AfuPV-2)	2
				Aspergillus fumigatus narnavirus 3 (AfuNV-3)	1 (partial)
6	V145-43	TR46/Y121F/T289A	resistant	Aspergillus fumigatus narnavirus 1 (AfuNV-1)	1
7	V145-80	none	susceptible	Aspergillus fumigatus narnavirus 2 (AfuNV-2)	1
8	V157-59	M220R	resistant	no virus found	NA
9	V108-20	none	susceptible	no virus found	NA
10	V157-80	P216L	resistant	Aspergillus fumigatus narnavirus 3 (AfuNV-3)	1 (partial)
11	V181-30	TR92/Y121F/T289A	resistant	Aspergillus fumigatus polymycovirus 1 (AfuPmV-1)	4

Mycelia were collected by centrifugation and transferred to a 1.5 mL tube containing 400–600 μm acid-washed glass beads. Mycelial cells were disrupted by three rounds of snap-freezing in liquid nitrogen and homogenization with a MagnaLyser (Roche) for 30 sec at 7,000 rpm. Total RNA was isolated with TRIzol reagent according to the protocol of the supplier (Life Technologies).

### RNA-sequencing

The mRNA library was constructed using the Illumina TruSeq RNA Sample Preparation v2 Guide (Illumina Inc., San Diego, USA) according to manufacturer’s instructions. The Low Sample (LS) protocol was used. Briefly, each RNA sample (1 μg) was enriched using oligo(dT)-tagged beads. RNA samples were fragmented into smaller pieces and used to synthesize cDNA. The library construction involved end repair, A-tailing, adapter ligation, and amplification. Mean length of each library was approximately 260 base pairs (bp). Sequencing was performed in a paired-end 2 x 75 bp mode on a NextSeq500 sequencer (Illumina®, Inc., San Diego, USA).

### Analysis of non-*Aspergillus fumigatus* reads

Illumina reads were mapped to the genome sequence of *A*. *fumigatus Af*293 CADRE 30 using CLC Genomics Workbench version (11.0). Unmapped reads were collected and used for *de novo* assembly using CLC Genomics Workbench version (11.0) with default settings.

Standalone Blast version 2.6.0 (Reference) was used to generate a Blast database containing all known mycovirus protein sequences. Mycovirus protein sequences were extracted from GenBank files and described in S1 Table. *De novo* derived contigs were analyzed in a blastx search using the mycovirus protein database.

### Phylogenetic analysis

For phylogenetic analysis, the RNA dependent RNA polymerase (RdRp) encoding regions of the contigs and the reference mycovirus sequences were aligned using CLC Genomics Workbench version 11.0 and maximum likelihood trees were constructed with 1000 bootstrap replicates.

## Results

### Mycovirus detection in *Aspergillus fumigatus* isolates

Transcriptomic analysis was performed on 11 different *A*. *fumigatus* strains (Table 1). Included were two sets of isogenic strains originating from the same patients. Both patients developed resistance during azole therapy. Isogenic isolates from patients 1 (strain 1, 8, and 10) showed different *CYP51A* mutations leading to azole resistance in strains 8 and 10. Isogenic isolates from patient 2 (strains 2 and 5) did not show changes in the *CYP51A* gene although strain 5 was azole resistant in contrast to strain 2. Six unrelated strains were isolated from the environment. Three of these strains (3, 6, and 11) contained mutations in the *CYP51A* gene resulting in azole resistance. The other three strain (4, 7, and 9) were susceptible to azole treatment. Sequencing reads were mapped against the genomic DNA sequence of *A*. *fumigatus Af*293 reference strain. Unmapped reads were collected and used for *de novo* assembly. Resulting contigs were analyzed with a standalone version of the Blastx program against a home-made database generated from known mycovirus protein sequences (S1 Table). In nine out of 11 strains, potential mycovirus sequences were detected. The results are shown in Table 1. Contigs encoding viral protein sequences were analyzed in more detail.

### *Aspergillus fumigatus* partitivirus genomic organization and phylogeny

Contigs generated from strains 2 and 5 sequencing reads were found positive for a novel partitivirus (Table 1). Two different contigs were obtained from each strain. Sequence analysis revealed that the corresponding contigs were similar in both strains. So, strains 2 and 5 contained similar viruses. One contig of 1822 bp represented a complete open reading frame (ORF) encoding an RNA dependent RNA polymerase (RdRp). Protein blast revealed highest similarity of 73% with RdRp of Alternaria alternata partitivirus-1. The other contig of 1638 bp represented an ORF encoding a protein with highest similar of 50% to the coat protein (CP) of Alternaria alternata partitivirus. The two contigs found, represented the two full genomic segments of a partitivirus.

**Fig 1:**
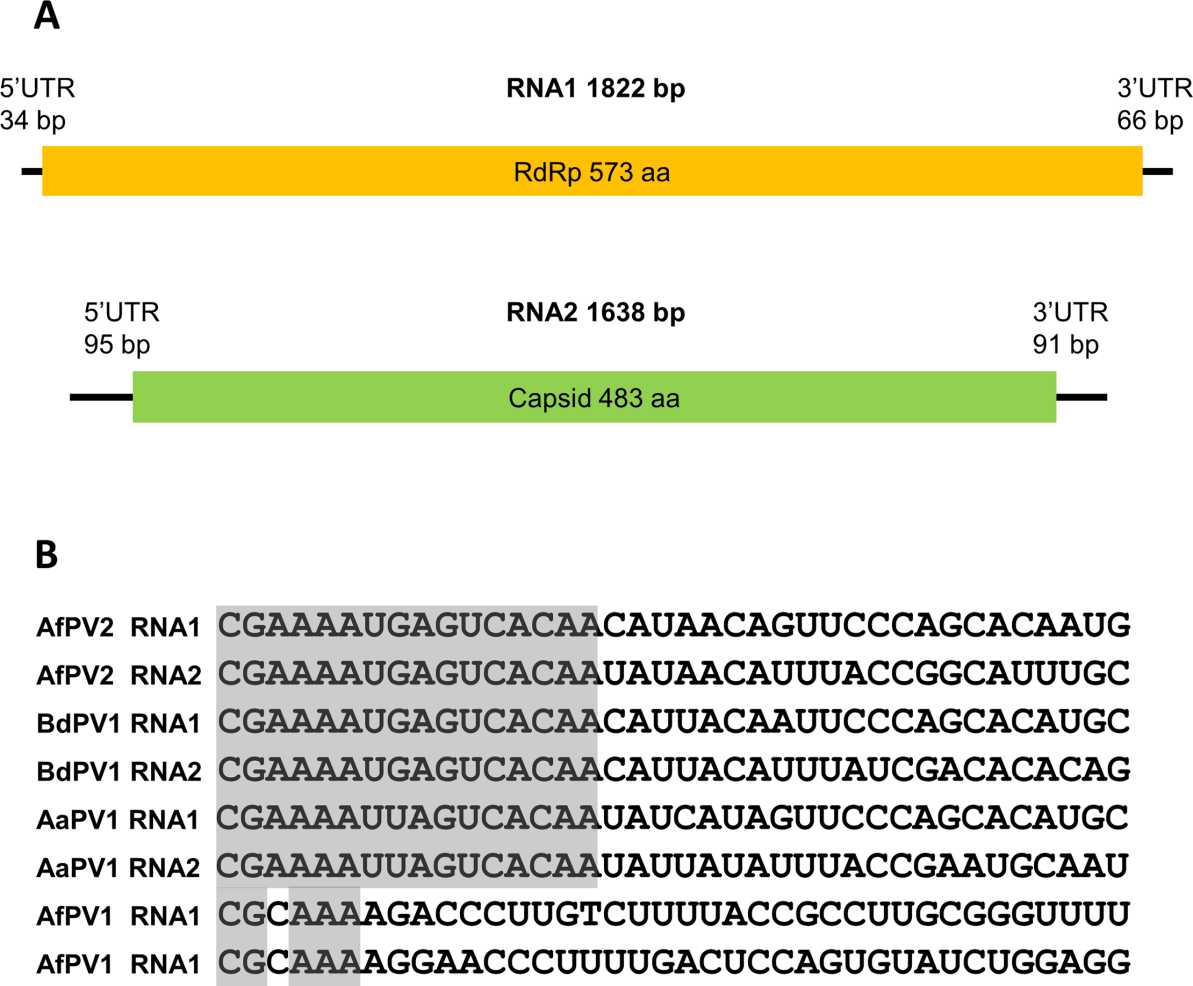
(A) Schematic representation of the genomic organization of the two segments of the novel Aspergillus fumigatus partitivirus-2 (AfuPV2). (B) Alignment of the 5’-UTR regions of related partitiviruses. Conserved nucleotides found in the genomic segments of Aspergillus fumigatus partitiviruses-1 and -2, Botryosphaeria dothidea partivirus-1, and Alternaria alternata partitivirus-1 are indicated.

The genomic organization of the newly found partitivirus segments is depicted in Fig 1A. The 1822 bp fragment contained an ORF encoding an RdRp protein of 573 amino acid residues. The closely related Alternaria alternata partitivirus-1 RdRp consists of 571 amino acids. The 1638 bp fragment encoded a protein of 483 amino acid residues, which is in the same range of the Alternaria alternata partitivirus-1 CP. The 5’-end nucleotide sequences of the contigs were aligned with the 5’-untranslated region (5’-UTR) of the two RNA segments of Alternaria alternata partitivirus-1, Botryosphaeria dothidea partitivirus-1, and Aspergillus fumigatus partitivirus-1 (Fig 1B). The extreme 16 nucleotide of the new partitivirus segments were highly similar to the corresponding regions of the first two partitiviruses. This indicates that the 5’-UTR of the new partitiviruses might be completely resolved.

**Fig 2:**
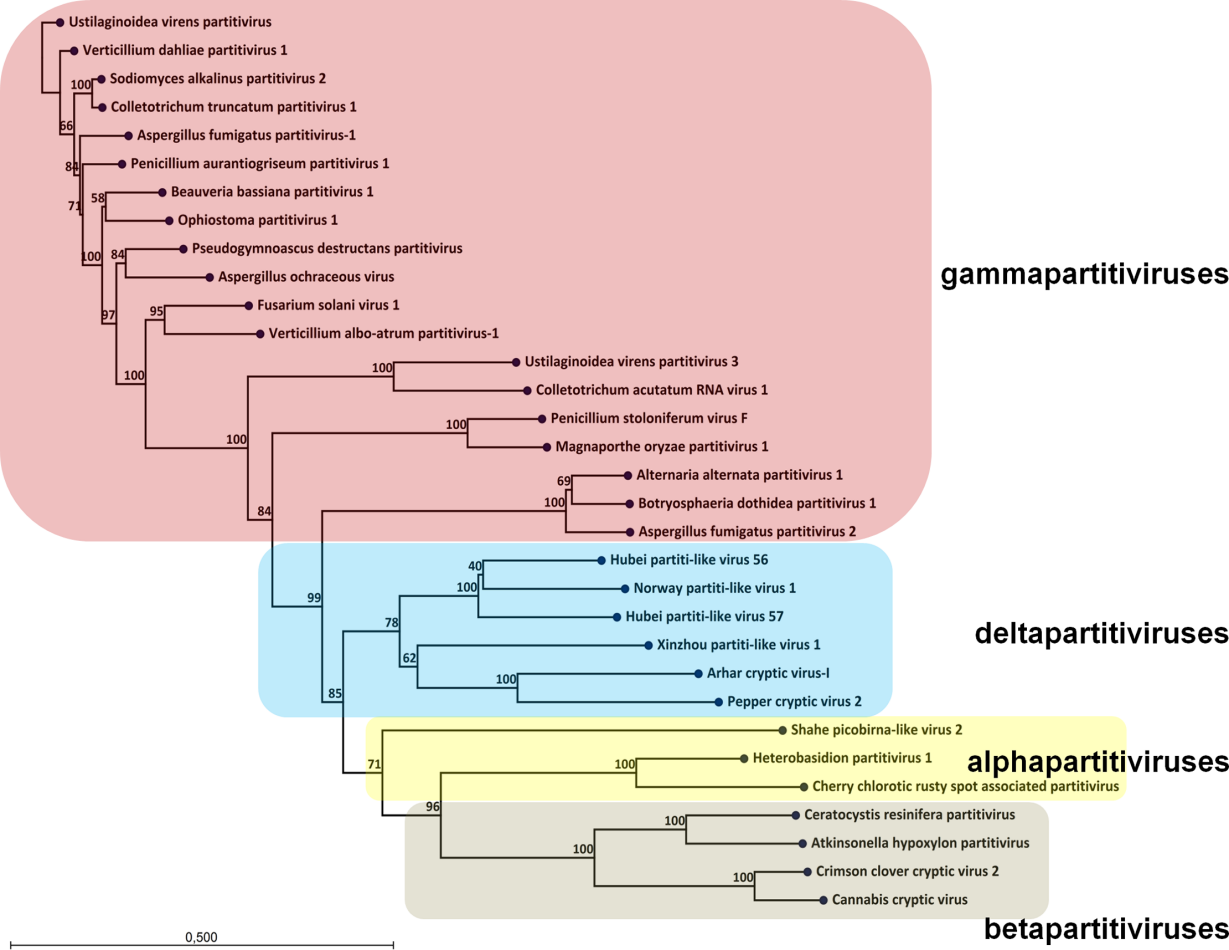
Phylogenetic relationships of the family partitiviridae. An unrooted phylogenetic tree was calculated from a multiple alignment of the RdRp protein using the maximum likelihood method with 1000 bootstrap replicates. The various subgenera are indicated by colored boxes.

Phylogenetic analysis was performed using RdRp protein sequences from alpha-, beta-, gamma-, and delta partitiviruses demonstrating a clustering of the newly found Aspergillus fumigatus partitivirus with Alternaria alternata partitivirus-1 and Botryosphaeria dothidea partitivirus-1 (Fig 2). The three viruses formed a separated clade between the groups of gamma- and delta-partitiviruses. The previously described Aspergillus fumigatu*s* partitivirus-1 appeared to be only distantly related to the newly found virus. Therefore, we propose the newly discovered virus to be named Aspergillus fumigatus partitivirus-2 (AfuPV-2). Nucleotide sequences of both segments are available in GenBank under accession numbers MH192991 and MH192992.

### *Aspergillus fumigatus* polymycovirus 1

From two out of 11 strains, multiple contigs were generated representing virus genomic segments of the previously described Aspergillus fumigatus polymycovirus-1 (AfuPmV-1) [12]. All four genomic segments were completely recovered and showed 95% to 98% similarity in nucleotide sequences.

The sequences of the newly found AfuPmV-1 variant are available in GenBank under accession numbers MH192993, MH192994, MH192995, and MH192996.

### *Aspergillus fumigatus narnaviridae*: Genomic organization and phylogeny

Contigs generated from seven *A*. *fumigatus* strains were found positive for novel narnaviruses (Table 1). A contig of 2007 bp was generated from strain-6 sequencing reads and represented a single ORF encoding an RdRp protein of 618 amino acid residues. Protein blast revealed an amino acid identity of 54% with the RdRp from Fusarium poae narnavirus-2. Contigs were generated from *A*. *fumigatus* strain 3 and 7 sequencing reads. Sequence alignment showed that both contig sequences were highly similar. Like the strain 6 contig, these 1994 bp segments contained a single ORF encoding narnavirus RdRp as well. Protein blast demonstrated an amino acid identity of 48% with Fusarium poae narnavirus-2. The two newly found Aspergillus fumigatus narnaviruses showed an amino acid sequence identity of 50%. Contigs generated from four different strains (1, 2, 5, and 10) demonstrated an amino acid sequence similarity of approximately 35% with Beihai narna-like virus-21. However, these segments appeared to be incomplete (data not shown).

**Fig 3:**
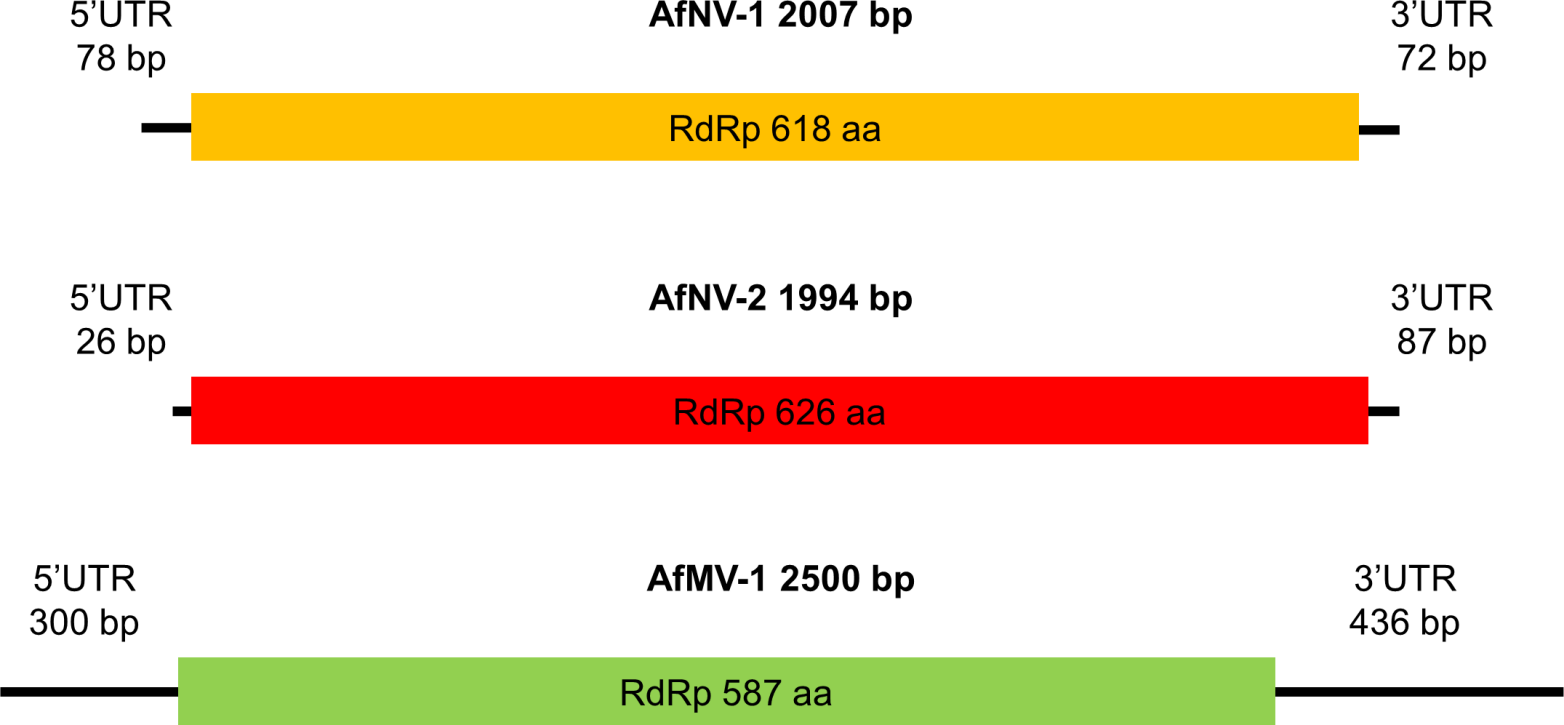
Schematic representation of the genomic organization of the novel narnaviridae Aspergillus fumigatus narnavirus-1 (AfuNV1), Aspergillus fumigatus narnavirus-2 (AfuNV2), and Aspergillus fumigatus mitovirus-1 (AfuMV).

Apart from the Aspergillus fumigatus narnavirus segment, an extra contig was generated from strain 3 reads. The 2500 bp fragment contained one single ORF encoding mitovirus RdRp. Blast analysis revealed that the protein had an amino acid sequence similarity of 31% with Sclerotina sclerotiorum ourmia-like virus-2. The genomic organization of the viral segments with complete protein coding regions are depicted in Fig 3.

**Fig 4:**
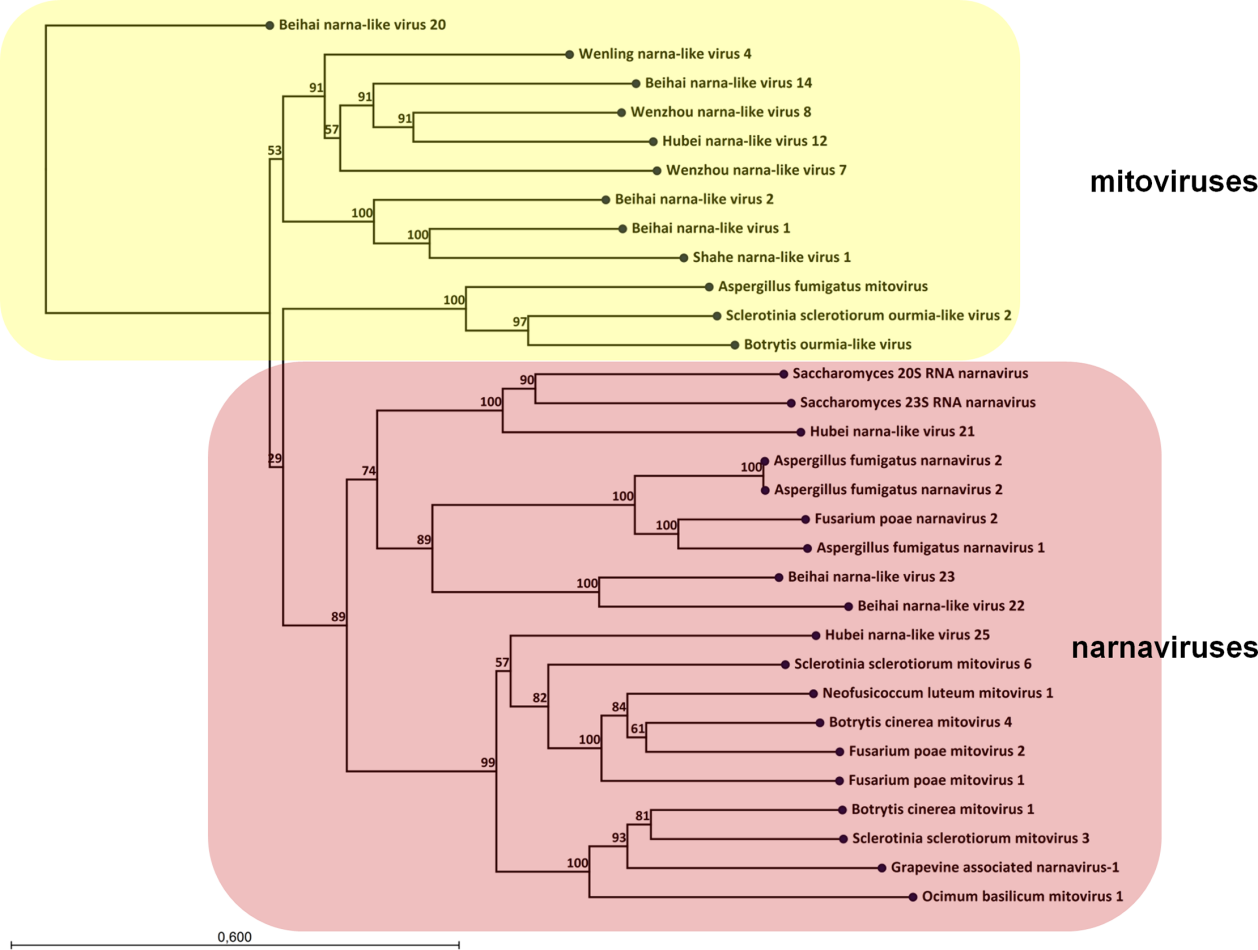
Phylogenetic relationships of the family Narnaviridae. An unrooted phylogenetic tree was calculated from a multiple alignment of the RdRp protein using the maximum likelihood method with 1000 bootstrap replicates. The various subgenera are indicated by colored boxes. The narnavirus and mitovirus genera are indicated.

Phylogenetic analysis was performed using RdRp sequences from plant and fungal narna- and mitoviruses and the newly found *A*. *fumigatus* narnaviruses and mitovirus (Fig 4). The analysis showed that the two *A*. *fumigatus* narnaviruses clustered with other known fungal narnaviruses, whereas the *A*. *fumigatus* mitovirus with known fungal mitoviruses. *A*. *fumigatus* narna- and mitoviruses were not previously described. We propose the newly found viruses to be named Aspergillus fumigatus narnavirus-1 (AfuNV-1), Aspergillus fumigatus narnavirus-2 (AfuNV-2), and Aspergillus fumigatus mitovirus-1 (AfuMV-1). Nucleotide sequences of the mitovirus and two narnaviruses are available in GenBank under accession numbers MH192988, MH192989, and MH192990, respectively.

## Discussion

Determination of the transcriptome results in vast amounts of RNA sequences from the cells of interest. Nearly all sequencing reads will be generated from cellular RNAs. However, the RNA content of a cell also includes parasitic molecules like RNA virus genomes and their replicative intermediates. In this paper, we describe the use of sequencing reads from transcriptomic experiments in *A*. *fumigatus* for the detection of RNA mycoviruses. The last few decades, new molecular methods gained more insight into the composition of the virosphere including mycoviruses. The number of RNA mycoviruses found in *A*. *fumigatus* so far, was limited to partitivirus, chrysovirus, and polymycovirus [12, 13]. The majority of mycovirus species was discovered by isolation and subsequent sequencing of dsRNA fragments from fungal extracts. In this way, dsRNA viral genomes and dsRNA replicative intermediates of ssRNA viruses could be recovered. However, recovery of dsRNA replicative intermediates from ssRNA viruses seems to be less efficient than dsRNA genomic RNA. This might be the reason for the underrepresentation of ssRNA mycovirus in several studies. Transcriptome analyses make use of both single stranded and double stranded RNA and this approach might be more sensitive for the detection and discovery of ssRNA mycoviruses. Indeed, in this study contigs were generated comprising *Narnaviridae* sequences from seven out of 11 strains. In total, nine out of 11 strains (81%) tested were infected with one or more mycovirus species. This is higher than reported by others [14, 15]. Several studies described the screening of different *Aspergillus* species for the presence of mycoviruses (reviewed by Kotta-Loizou and Coutts) [7]. The percentages of infected isolates ranged from 7% to 50%. In contrast to other studies, we did not detect chrysovirus in our *A*. *fumigatus* strains. Bhatti *et al*. found dsRNA segments in 6.6% of 366 *A*. *fumigatus* strains tested. Only 5 (1.4%) of these strain were infected with chrysovirus [14]. The number of dsRNA virus infected isolates in this study was four out of 11 (36%), which was in the range of infected isolates mentioned in earlier studies as described above. The absence of chrysovirus in our samples might be due to the limited number of strains used in this study. Until now, only dsRNA mycoviruses were detected in aspergilli [7]. This demonstrates the benefit of our approach in detecting ssRNA mycoviruses.

Two previous studies reported the presence of partitivirus in *Aspergillus* [13, 16]. These viruses, Aspergillus ochraceus virus (Aov) and Aspergillus fumigatus partitivirus-1 (AfuPV-1) are both members of the *Gammapartitivirus* genus which includes many fungal specific partitiviruses. In this study, we found a new partitivirus, Aspergillus fumigatus partitivirus-2 (AfuPV-2). Phylogenetic analysis showed that AfuPV-2 is related to Alternaria alternata partitivirus-1 and Botryosphaeria dothidea partitivirus-1 [17, 18]. These three viruses form a phylogenetic clade within the *Gammapartitivirus* genus but the clade might be considered as an intermediate between the *Gammapartitivirus* and *Deltapartitivirus* genera. Xavier *et al*. described genus specific sequences in the 5’-UTR of partitiviruses [18]. The *Gammapartitivirus* specific element is found in Aspergillus fumigatus partitivirus-2 5’-UTR as well (Fig 1B).

The Aspergillus fumigatus polymycovirus-1 (AfuPmV-1) has been detected in a single occasion [12]. This virus was originally named Aspergillus fumigatus tetramycovirus (AfuTMV) since the dsRNA genome is divided over four genomic segments. AfuPmV-1 is the prototype species of the proposed *Polymycoviridae* family [19]. In this study, two unrelated *A*. *fumigatus* isolates were found to be infected with AfuPmV-1. All four genomic segments could be retrieved from the sequence data. Multiple sequence alignment showed a high degree of similarity between the newly found genomes and the previously described viral segments from 95% to 98% at the nucleotide level.

Members of the Narnaviridae family belong to a group of viruses without a coat protein encoding gene. Consequently, the naked RNA viruses (*Narnaviridae*) are not encapsidated [4]. The unsegmented genome only contains an RdRp encoding open reading frame. The *Narnaviridae* family includes the *Narnavirus* and *Mitovirus* genera. Mitoviruses are thought to reside exclusively in the mitochondria, whereas Narnaviruses can replicate in the cytoplasm of the infected host [4]. *Narnaviridae* forms infectious agents replicating in plants and fungi. Until now, no *Narnaviridae* were detected in *Aspergillus*. Here, we report the nucleotide sequences of two *Aspergillus fumigatus* narnaviruses and one mitovirus which are related to known fungal *Narnaviridae*.

The role of mycovirus infections in fungal virulence is still under debate. Hypovirulence effect of mycovirus infections in a number of phytopathogenic fungi were previously described (Reviewed in Xie and Jiang 2014) [20]. However, it is unclear if the same effects can be achieved in human pathogenic fungi like *A*. *fumigatus*. Induction of hypovirulence by virus infections is of special interest for human fungal pathogens. The attenuating effect of mycovirus infection might be a potential tool for the treatment of fungal infections in agriculture as well as in human health. In the last decade, emergence of pan-azole resistance *A*. *fumigatus* strains has been described. Pan-azole resistance has major consequences for the treatment of patients with invasive aspergillosis and causes a subsequent increase in mortality rate up to 90% [21, 22]. Due to the lack of therapeutic drugs, alternative treatments might become more important. Although there is no relationship between mycovirus infection and the development of azole resistance, mycovirus therapy might be helpful as an alternative treatment of fungicide resistant fungi [23–27]. However, experimental design is hampered by the lack of extracellular infectious virus particles. Therapeutic usage of mycoviruses in fungal infections is therefore not very obvious.

The number of viruses infecting filamentous fungi seems to be underestimated so far. Until now, only one single stranded DNA mycovirus was detected [8]. No double stranded DNA mycovirus were ever recognized. Use of advanced molecular techniques will be necessary to reveal the complete mycovirus landscape.

## Supporting information

S1 Table Mycovirus protein sequences used in the reference database. (PDF)
