# Supplementary material for: Discovery and characterization of novel Aspergillus fumigatus mycoviruses
Source: PLoS One. 2018 Jul 25;13(7):e0200511. doi: 10.1371/journal.pone.0200511 (PMC6059430; doi:10.1371/journal.pone.0200511)
Supplement: S1 Table — (PDF) [file pone.0200511.s001.pdf]

virus family	virus genus	virus species	genomic (segment) RNA length (bp)	Accession number
Alphaflexiviridae	Botrexvirus	Botrytis virus X	6966	NC_005132
	Sclerodarnavirus	Sclerotinia sclerotiorum debilitation-associated RNA virus	5470	NC_007415
Deltaflexiviridae	Deltaflexivirus	Fusarium graminearum deltaflexivirus 1	8246	NC_030654
Gammaflexiviridae	Mycoflexivirus	Botrytis virus F	6827	NC_002604
Barnaviridae	Barnavirus	Mushroom bacilliform virus	4009	NC_001633
		Rhizoctonia solani barnavirus 1	3914	KP900904
Chrysoviridae	Chrysovirus	Amasya cherry disease associated chrysovirus segment 1	3399	NC_009947
		Amasya cherry disease associated chrysovirus segment 2	3128	NC_009946
		Amasya cherry disease associated chrysovirus segment 3	2833	NC_009945
		Amasya cherry disease associated chrysovirus segment 4	2498	NC_009944
		Anthurium mosaic-associated virus RNA1	3550	FJ899675
		Anthurium mosaic-associated virus RNA2	3448	FJ899676
		Anthurium mosaic-associated virus RNA3	3244	FJ899677
		Aspergillus fumigatus chrysovirus segment 1	3560	FN178512
		Aspergillus fumigatus chrysovirus segment 2	3159	FN178513
		Aspergillus fumigatus chrysovirus segment 3	3006	FN178514
		Aspergillus fumigatus chrysovirus segment 4	2863	FN178515
		Bipolaris maydis chrysovirus 1	3617	KY489954
		Bipolaris maydis chrysovirus 2	3123	KY489955
		Bipolaris maydis chrysovirus 3	2984	KY489956
		Bipolaris maydis chrysovirus 4	2753	KY489957
		Botryosphaeria dothidea chrysovirus 1 RNA 1	3654	KF688736
		Botryosphaeria dothidea chrysovirus 1 RNA 2	2773	KF688737
		Botryosphaeria dothidea chrysovirus 1 RNA 3	2597	KF688738
		Botryosphaeria dothidea chrysovirus 1 RNA 4	2574	KF688739
		Botryosphaeria dothidea chrysovirus 1 segment 1	3528	NC_034268
		Botryosphaeria dothidea chrysovirus 1 segment 2	2816	NC_034271
		Botryosphaeria dothidea chrysovirus 1 segment 3	2583	NC_034269
		Botryosphaeria dothidea chrysovirus 1 segment 4	2574	NC_034270
		Botryosphaeria dothidea chrysovirus RNA 1	3655	KJ573505
		Botryosphaeria dothidea chrysovirus RNA 2	2825	KJ573506
		Botryosphaeria dothidea chrysovirus RNA 3	2596	KJ573507
		Botryosphaeria dothidea chrysovirus RNA 4	2575	KJ573508
		Brassica campestris chrysovirus 1 RNA 1	3639	KP782031
		Brassica campestris chrysovirus 1 RNA 2	3567	KP782030
		Brassica campestris chrysovirus 1 RNA 3	3337	KP782029
		Colletotrichum gloeosporioides chrysovirus 1 segment 1	3397	KT581957
		Colletotrichum gloeosporioides chrysovirus 1 segment 2	2869	KT581958

virus family	virus genus	virus species	genomic (segment) RNA length (bp)	Accession number
		Colletotrichum gloeosporioides chrysovirus 1 segment 3	2630	KT581959
		Cryphonectria nitschkei chrysovirus 1 BS122 CP	2980	GQ290645
		Cryphonectria nitschkei chrysovirus 1 BS122 RdRp	2978	GQ290649
		Cryphonectria nitschkei chrysovirus 1 BS131 CP	2718	GQ290646
		Cryphonectria nitschkei chrysovirus 1 BS131 RdRp	2889	GQ290650
		Cryphonectria nitschkei chrysovirus 1 BS132 CP	2718	GQ290647
		Cryphonectria nitschkei chrysovirus 1 BS132 RdRp	2889	GQ290651
		Cryphonectria nitschkei chrysovirus 1 BS321 CP	2721	GQ290648
		Cryphonectria nitschkei chrysovirus 1 BS321 RdRp	2889	GQ290652
		Cryphonectria nitschkei chrysovirus 1 segment 1	2960	HM013826
		Cryphonectria nitschkei chrysovirus 1 segment 2	2552	HM013825
		Fusarium oxysporum f. sp. dianthi mycovirus 1 segment 1	3555	NC_027563
		Fusarium oxysporum f. sp. dianthi mycovirus 1 segment 2	2809	NC_027564
		Fusarium oxysporum f. sp. dianthi mycovirus 1 segment 3	2794	NC_027565
		Fusarium oxysporum f. sp. dianthi mycovirus 1 segment 4	2646	NC_027566
		Grapevine chrysovirus protease	2762	JX658567
		Helminthosporium victoriae 145S virus RNA 1	3612	NC_005978
		Helminthosporium victoriae 145S virus RNA 2	3134	NC_005979
		Helminthosporium victoriae 145S virus RNA 3	3972	NC_005980
		Helminthosporium victoriae 145S virus RNA 4	2763	NC_005981
		Isaria javanica chrysovirus 1 segment 1	3593	NC_033277
		Isaria javanica chrysovirus 1 segment 2	3175	NC_033317
		Isaria javanica chrysovirus 1 segment 3	3165	NC_033318
		Isaria javanica chrysovirus 1 segment 4	2874	NC_033278
		La France disease virus ORF1	503	U62640
		La France disease virus ORF1	1101	U62643
		La France disease virus pL3 gene	2778	D10830
		La France disease virus pM1 gene	1407	D10829
		La France disease virus pM2 gene	1307	D10828
		Macrophomina phaseolina chrysovirus 1 RNA 1	3712	KP900886
		Macrophomina phaseolina chrysovirus 1 RNA 2	3462	KP900887
		Macrophomina phaseolina chrysovirus 1 RNA 3	2985	KP900889
		Macrophomina phaseolina chrysovirus 1 RNA 4	2927	KP900888
		Magnaporthe oryzae chrysovirus 1 segment 1	3554	NC_014462
		Magnaporthe oryzae chrysovirus 1 segment 2	3250	NC_014465
		Magnaporthe oryzae chrysovirus 1 segment 3	3074	NC_014463
		Magnaporthe oryzae chrysovirus 1 segment 4	3043	NC_014464
		Magnaporthe oryzae chrysovirus 1 segment 5	2879	AB700631

virus family	virus genus	virus species	genomic (segment) RNA length (bp)	Accession number
		Magnaporthe oryzae chrysovirus 3 RNA 1	3558	NC_023041
		Magnaporthe oryzae chrysovirus 3 RNA 2	3254	NC_023042
		Magnaporthe oryzae chrysovirus 3 RNA 3	2997	NC_023039
		Magnaporthe oryzae chrysovirus 3 RNA 4	2880	NC_023040
		Magnaporthe oryzae chrysovirus 3 RNA 5	2874	NC_023043
		Penicillium chrysogenum virus segment 1	3562	NC_007539
		Penicillium chrysogenum virus segment 2	3200	NC_007540
		Penicillium chrysogenum virus segment 3	2976	NC_007541
		Penicillium chrysogenum virus segment 4	2902	NC_007542
		Penicillium janczewskii chrysovirus 1 RNA 1	3698	NC_028495
		Penicillium janczewskii chrysovirus 1 RNA 2	2899	NC_028500
		Penicillium janczewskii chrysovirus 1 RNA 3	2942	NC_028496
		Penicillium janczewskii chrysovirus 1 RNA 4	2506	NC_028497
		Penicillium janczewskii chrysovirus 2 RNA 1	3540	KT950836
		Penicillium janczewskii chrysovirus 2 RNA 2	2699	KT950837
		Penicillium janczewskii chrysovirus 2 RNA 3	2535	KT950838
		Persea americana chrysovirus RNA 1	3421	KJ418374
		Persea americana chrysovirus RNA 2	3335	KJ418375
		Persea americana chrysovirus RNA 3	2857	KJ418376
		Raphanus sativas chrysovirus 1 RNA 1	3638	JQ045335
		Raphanus sativas chrysovirus 1 RNA 2	3517	JQ045336
		Raphanus sativas chrysovirus 1 RNA 3	3299	JQ045337
		Verticillium dahliae chrysovirus 1 segment 1	3594	HM004067
		Verticillium dahliae chrysovirus 1 segment 2	3313	HM004068
		Verticillium dahliae chrysovirus 1 segment 3	2983	HM004069
		Verticillium dahliae chrysovirus 1 segment 4	2932	HM004070
Endornaviridae	Alphaendornavirus	Basella alba alphaendornavirus 1	14027	AB844264
		Basella alba alphaendornavirus 1	14027	AB844265
		Bell pepper endornavirus	14727	AB597230
		Bell pepper endornavirus	14729	KT149366
		Bell pepper endornavirus	14728	NC_015781
		Bell pepper endornavirus	14727	JQ951943
		Bell pepper endornavirus	14727	KF709944
		Bell pepper endornavirus	14659	KP455654
		Bell pepper endornavirus	14727	KX977568
		Bell pepper endornavirus	14714	KX977569
		Cucumis melo endornavirus	15079	KX641269
		Cucumis melo endornavirus	15078	NC_029064

virus family	virus genus	virus species	genomic (segment) RNA length (bp)	Accession number
		Erysiphe cichoracearum endornavirus	11907	NC_029095
		Grapevine endophyte endornavirus	12154	NC_019493
		Helicobasidium mompa endornavirus 1	16614	NC_013447
		Hordeum vulgare endornavirus	14243	NC_028949
		Hot pepper endornavirus	14729	NC_027920
		Lagenaria siceraria endornavirus-California	15088	NC_023641
		Oryza rufipogon endornavirus	13936	NC_007649
		Oryza sativa endornavirus	13952	NC_007647
		Persea americana alphaendornavirus 1	13459	NC_016648
		Phaseolus vulgaris endornavirus 1	14072	KT456287
		Phaseolus vulgaris endornavirus 1	13908	NC_023678
		Phaseolus vulgaris endornavirus 2	14820	AB719398
		Phaseolus vulgaris endornavirus 2	14817	KT456288
		Phytophthora endornavirus 1	13883	NC_007069
		Rhizoctonia cerealis endornavirus 1	17486	NC_022619
		Vicia faba endornavirus	17635	NC_007648
		Yerba mate endornavirus	13954	NC_024455
	Betaendornavirus	Alternaria brassicicola endornavirus 1	10290	NC_026136
		Gremmeniella abietina type B RNA virus XL1	10375	NC_007920
		Sclerotinia sclerotiorum endornavirus 2	10520	KU299046
		Sclerotinia sclerotiorum endornavirus 2	10513	NC_023893
		Tuber aestivum endornavirus	9760	NC_014904
	unclassified	Botrytis cinerea endornavirus 1	11557	NC_031752
		Ceratobasidium endornavirus A	15207	NC_031462
		Ceratobasidium endornavirus B	23635	NC_031463
		Ceratobasidium endornavirus C	21004	NC_031461
		Ceratobasidium endornavirus D	19406	NC_031449
		Ceratobasidium endornavirus G	19293	NC_031464
		Lagenaria siceraria endornavirus-Hubei	15098	NC_034216
		Psophocarpus tetragonolobus endornavirus	14623	NC_031336
		Rhizoctonia solani endornavirus 2	15850	KT823701
		Rosellinia necatrix endornavirus 1	9639	NC_030938
		Sclerotinia sclerotiorum endornavirus-1	10580	KM923990
		Sclerotinia sclerotiorum endornavirus-1	10770	NC_021706
Hypoviridae	Hypovirus	Cryphonectria hypovirus 1	12727	AF082191
		Cryphonectria hypovirus 1	12724	DQ861913
		Cryphonectria hypovirus 1	3234	HM246637
		Cryphonectria hypovirus 1	3231	HM246638

virus family	virus genus	virus species	genomic (segment) RNA length (bp)	Accession number
		Cryphonectria hypovirus 1	3237	HM246639
		Cryphonectria hypovirus 1	3231	HM246640
		Cryphonectria hypovirus 1	3234	HM246641
		Cryphonectria hypovirus 1	3234	HM246642
		Cryphonectria hypovirus 1	3237	HM246643
		Cryphonectria hypovirus 1	3234	HM246644
		Cryphonectria hypovirus 1	3234	HM246645
		Cryphonectria hypovirus 1	3234	HM246646
		Cryphonectria hypovirus 1	3237	HM246647
		Cryphonectria hypovirus 1	3232	HM246648
		Cryphonectria hypovirus 1	2769	HM246649
		Cryphonectria hypovirus 1	3237	HM246650
		Cryphonectria hypovirus 1	12730	KT726153
		Cryphonectria hypovirus 1	12734	NC_001492
		Cryphonectria hypovirus 3	9799	NC_000960
		Cryphonectria hypovirus 3 (CS)	9591	AF188514
		Cryphonectria hypovirus 3 RNA2	3634	AF276810
		Cryphonectria hypovirus 4	9149	NC_006431
		Fusarium graminearum hypovirus 1	13023	NC_023680
		Fusarium graminearum hypovirus 2	4553	KP208179
		Fusarium graminearum hypovirus 2	12800	NC_026813
		Fusarium langsethiae hypovirus 1	12839	NC_032212
		Fusarium poae hypovirus 1	12795	LC150612
		Grapevine associated hypovirus-1	853	GU108593
		Phomopsis longicolla hypovirus	9760	NC_024685
		Sclerotinia sclerotiorum hypovirus 1	3657	JN084008
		Sclerotinia sclerotiorum hypovirus 1	10438	NC_015939
		Sclerotinia sclerotiorum hypovirus 2	14580	KF898354
		Sclerotinia sclerotiorum hypovirus 2	15239	KJ561218
		Sclerotinia sclerotiorum hypovirus 2	14581	NC_022896
		Trichoderma hypovirus	1923	JX291540
		Valsa ceratosperma hypovirus 1	9543	NC_017099
Narnaviridae	Mitovirus	Alternaria arborescens mitovirus 1	2506	NC_030747
		Alternaria brassicicola mitovirus	2506	KP705075
		Binucleate Rhizoctonia mitovirus K1	2794	NC_027921
		Botrytis cinerea mitovirus 1	2804	NC_011372
		Botrytis cinerea mitovirus 1 S	2171	EF583556
		Botrytis cinerea mitovirus 2	2497	NC_028471

virus family	virus genus	virus species	genomic (segment) RNA length (bp)	Accession number
		Botrytis cinerea mitovirus 3	2922	NC_028472
		Botrytis cinerea mitovirus 4	2768	NC_028474
		Buergenerula spartinae mitovirus 1	2735	KJ485703
		Clitocybe odora virus	3765	NC_017003
		Cronartium ribicola mitovirus 1	2715	NC_030393
		Cronartium ribicola mitovirus 2	2471	NC_030395
		Cronartium ribicola mitovirus 3	2522	NC_030396
		Cronartium ribicola mitovirus 4	2479	NC_030397
		Cronartium ribicola mitovirus 5	2631	NC_030399
		Cryphonectria cubensis mitovirus 1a	2555	AY328476
		Cryphonectria cubensis mitovirus 1b	2601	AY328477
		Cryphonectria cubensis mitovirus 1c	2501	AY328478
		Cryphonectria cubensis mitovirus 2a	2639	AY328479
		Cryphonectria cubensis mitovirus 2c	2419	AY328481
		Cryphonectria parasitica mitovirus 1-NB631	2728	NC_004046
		Fusarium circinatum mitovirus 1	2419	KF803546
		Fusarium circinatum mitovirus 2-1	2193	KF803547
		Fusarium coeruleum mitovirus 1	2423	NC_026622
		Fusarium globosum mitovirus 1	2414	NC_026621
		Fusarium poae mitovirus 1	2397	NC_030861
		Fusarium poae mitovirus 2	2414	NC_030862
		Fusarium poae mitovirus 3	2718	NC_030863
		Fusarium poae mitovirus 4	2387	NC_030864
		Gremmeniella abietina mitochondrial RNA virus S2	2587	NC_006264
		Gremmeniella abietina mitovirus S1	2572	AF534641
		Gremmeniella abietina non-host-specific mitochondrial RNA virus S1	2544	JN654496
		Helicobasidium mompa mitovirus 1-18 RDRP gene	2411	AB110977
		Heterobasidion mitovirus 1	4379	KJ873059
		Hymenoscyphus fraxineus mitovirus 1	2387	KJ667051
		Hymenoscyphus fraxineus mitovirus 1	2350	KT809403
		Macrophomina phaseolina mitovirus 1	2572	KP900894
		Macrophomina phaseolina mitovirus 3	4179	KT823703
		Mitovirus AEF-2013	2550	KF298270
		Mitovirus AEF-2013	2486	KF298281
		Neofusicoccum luteum mitovirus 1	2389	NC_035114
		Ophiostoma mitovirus 1c	3107	KF026355
		Ophiostoma mitovirus 3a	2617	NC_004049
		Ophiostoma mitovirus 4	2599	NC_004052

virus family	virus genus	virus species	genomic (segment) RNA length (bp)	Accession number
		Ophiostoma mitovirus 5	2474	NC_004053
		Ophiostoma mitovirus 6	2343	NC_004054
		Ophiostoma mitovirus 7	2804	KF031943
		Rhizoctonia cerealis mitovirus	3149	KM517201
		Rhizoctonia mitovirus 1 RS002	2797	KC792591
		Rhizoctonia mitovirus 1 RS006-2	2779	KC792598
		Rhizoctonia oryzae-sativae mitovirus 1	3038	NC_029991
		Rhizoctonia solani mitovirus 11	3283	KP900906
		Rhizoctonia solani mitovirus 12	3378	KP900907
		Rhizoctonia solani mitovirus 13	3039	KP900908
		Rhizoctonia solani mitovirus 15	3901	KP900910
		Rhizoctonia solani mitovirus 2	2865	KP900911
		Rhizoctonia solani mitovirus 6	2615	KP900915
		Rhizoctonia solani mitovirus 8	3225	KP900917
		Rhizophagus sp. HR1 mitovirus	3668	AB855794
		Rhizophagus sp. RF1 mitovirus	2895	AB558120
		Sclerotinia homoeocarpa mitovirus	2632	AY172454
		Sclerotinia nivalis mitovirus 1	2720	KT365895
		Sclerotinia nivalis mitovirus 2	2583	KT365896
		Sclerotinia sclerotiorum mitovirus 1	2513	JQ013377
		Sclerotinia sclerotiorum mitovirus 1 HC025	2530	NC_026510
		Sclerotinia sclerotiorum mitovirus 11	2708	KF913886
		Sclerotinia sclerotiorum mitovirus 12	2593	KF913887
		Sclerotinia sclerotiorum mitovirus 14	2564	KF913889
		Sclerotinia sclerotiorum mitovirus 15	2548	KF913890
		Sclerotinia sclerotiorum mitovirus 17	2447	KP900924
		Sclerotinia sclerotiorum mitovirus 18	2509	KP900925
		Sclerotinia sclerotiorum mitovirus 19	3185	KP900926
		Sclerotinia sclerotiorum mitovirus 2	2445	JQ013378
		Sclerotinia sclerotiorum mitovirus 2	2438	JX401536
		Sclerotinia sclerotiorum mitovirus 2	2438	KJ462508
		Sclerotinia sclerotiorum mitovirus 20	2648	KP900927
		Sclerotinia sclerotiorum mitovirus 3	2588	JX401537
		Sclerotinia sclerotiorum mitovirus 3	2617	NC_028475
		Sclerotinia sclerotiorum mitovirus 4	2744	JX401538
		Sclerotinia sclerotiorum mitovirus 4	2752	KT962974
		Sclerotinia sclerotiorum mitovirus 5	2498	KJ462509
		Sclerotinia sclerotiorum mitovirus 5	2498	KJ462510

virus family	virus genus	virus species	genomic (segment) RNA length (bp)	Accession number
		Sclerotinia sclerotiorum mitovirus 5	2497	KJ462511
		Sclerotinia sclerotiorum mitovirus 6	2535	KF913881
		Sclerotinia sclerotiorum mitovirus 6	2584	KJ462512
		Sclerotinia sclerotiorum mitovirus 6	2586	KJ462513
		Sclerotinia sclerotiorum mitovirus 6	2563	NC_023598
		Sclerotinia sclerotiorum mitovirus 7	2648	KF913882
		Sclerotinia sclerotiorum mitovirus 7	2815	KJ462514
		Sclerotinia sclerotiorum mitovirus 8	2565	KF913883
		Sclerotinia sclerotiorum mitovirus 9	2562	KF913884
		Thielaviopsis basicola mitovirus	2896	NC_012585
		Tuber aestivum mitovirus	3480	NC_015629
		Tuber excavatum mitovirus	3305	JN222389
	Narnavirus	Fusarium poae narnavirus 1	2297	NC_030865
		Fusarium poae narnavirus 2	2054	NC_030866
		Grapevine associated narnavirus-1	622	GU108590
		Grapevine associated narnavirus-1	2733	NC_028473
		Leptomonas seymouri Narna-like virus 1 segment L	2914	KU935604
		Leptomonas seymouri Narna-like virus 1 segment S	1455	KU935605
		Phytomonas serpens narnavirus 1	3782	NC_030308
		Saccharomyces 20S RNA narnavirus	2514	NC_004051
		Saccharomyces 23S RNA narnavirus	2891	NC_004050
	unclassified	Diatom colony associated dsRNA virus 2	4586	AP014913
Partitiviridae	Alphapartitivirus	Helicobasidium mompa partitivirus V1-1 RDRP gene	2247	AB110979
		Helicobasidium mompa partitivirus V1-2 RDRP gene	1776	AB110980
		Heterobasidion RNA virus 1 CP	1866	HQ541324
		Heterobasidion RNA virus 1 RdRp	2027	HQ541323
		Heterobasidion RNA virus 1 RdRp	2029	HQ541328
		Heterobasidion RNA virus 1 RdRp	2033	HQ541329
		Heterobasidion RNA virus 1 RdRp	2030	HQ541330
		Heterobasidion RNA virus 1 RdRp	2028	HQ541332
		Heterobasidion RNA Virus 3 CP	1826	FJ816272
		Heterobasidion RNA Virus 3 RdRp	1885	FJ816271
		Rosellinia necatrix partitivirus 2 CP	1828	NC_020235
		Rosellinia necatrix partitivirus 2 RdRp	1985	NC_020234
	Betapartitivirus	Atkinsonella hypoxylon partitivirus RNA 1	2180	NC_003470
		Atkinsonella hypoxylon partitivirus RNA 2	2135	NC_003471
		Cannabis cryptic virus CP	2290	JN196537
		Cannabis cryptic virus CP	2266	NC_031130

virus family	virus genus	virus species	genomic (segment) RNA length (bp)	Accession number
		Cannabis cryptic virus RdRp	2420	JN196536
		Cannabis cryptic virus RdRp	2397	NC_031134
		Ceratocystis resinifera virus 1 RNA 1	2207	NC_010755
		Ceratocystis resinifera virus 1 RNA 2	2305	NC_010754
		Crimson clover cryptic virus 2 RNA 1	2444	JX971982
		Crimson clover cryptic virus 2 RNA 2	2354	JX971983
		Cucurbitaria piceae virus 1 segment 1	2318	KT343866
		Dill cryptic virus 2 RNA 1	2430	NC_021147
		Dill cryptic virus 2 RNA 2	2354	NC_021148
		Fusarium poae virus 1 RNA 1	2185	NC_003883
		Fusarium poae virus 1 RNA 2	2203	NC_003884
		Fusarium poae virus 1-240374 segment 1	2431	NC_030882
		Fusarium poae virus 1-240374 segment 2	2231	NC_030877
		Heterobasidion annosum P-type partitivirus RdRp	2325	AF473549
		Heterobasidion partitivirus 17 RdRp	2016	KJ873060
		Heterobasidion partitivirus 2 CP	2238	HM565954
		Heterobasidion partitivirus 2 RdRp	2290	HM565953
		Heterobasidion partitivirus 8 CP	2235	JX625228
		Heterobasidion partitivirus 8 RdRp	2281	JX625227
		Hop trefoil cryptic virus 2 RNA 1	2431	NC_021098
		Hop trefoil cryptic virus 2 RNA 2	2349	NC_021099
		Lentinula edodes partitivirus 1 CP	2245	KX354972
		Lentinula edodes partitivirus 1 RdRp	2382	KX354971
		Pleurotus ostreatus virus 1 RNA-1	2296	NC_006961
		Pleurotus ostreatus virus 1 RNA-2	2223	NC_006960
		Primula malacoides virus China/Mar2007 RNA 1	2390	NC_013109
		Primula malacoides virus China/Mar2007 RNA 2	2344	NC_013110
		Red clover cryptic virus 2 RNA 1	2430	NC_021096
		Red clover cryptic virus 2 RNA 2	2353	NC_021097
		Rhizoctonia solani virus 717 RNA 1	1869	KM657430
		Rhizoctonia solani virus 717 RNA 2	1833	KM657431
		Rhizoctonia solani virus 717 RNA1	2363	NC_003801
		Rhizoctonia solani virus RNA2	2206	NC_003802
		Rosellinia necatrix partitivirus 1-W8 segment 1	2299	NC_007537
		Rosellinia necatrix partitivirus 1-W8 segment 2	2279	NC_007538
		Rosellinia necatrix partitivirus 6 CP	2499	NC_028250
		Rosellinia necatrix partitivirus 6 RdRp	1946	LC062720
		Rosellinia necatrix partitivirus 6 RdRp	2204	LC062721

virus family	virus genus	virus species	genomic (segment) RNA length (bp)	Accession number
		Rosellinia necatrix partitivirus 6 RdRp	2266	LC062722
		Rosellinia necatrix partitivirus 6 RdRp	2047	LC062723
		Rosellinia necatrix partitivirus 6 RdRp	2076	LC062724
		Rosellinia necatrix partitivirus 6 RdRp	2254	LC062725
		Rosellinia necatrix partitivirus 6 RdRp	1979	LC062726
		Rosellinia necatrix partitivirus 6 RdRp	1808	LC062727
		Rosellinia necatrix partitivirus 6 RdRp	2462	NC_028251
		White clover cryptic virus 2 RNA 1	2435	NC_021094
		White clover cryptic virus 2 RNA 2	2348	NC_021095
	Gammapartitivirus	Aspergillus ochraceous virus CP	1555	EU118278
		Aspergillus ochraceous virus RdRp	1754	DQ270031
		Aspergillus ochraceous virus RdRp	1754	EU118277
		Discula destructiva virus 1 segment 1	1787	NC_002797
		Discula destructiva virus 1 segment 2	1585	NC_002800
		Discula destructiva virus 2 segment 1	1781	NC_003710
		Discula destructiva virus 2 segment 2	1611	NC_003711
		Fusarium solani virus 1 RNA 1	1645	NC_003885
		Fusarium solani virus 1 RNA 2	1445	NC_003886
		Gremmeniella abietina RNA virus MS1 RNA 1	1781	KJ786413
		Gremmeniella abietina RNA virus MS1 RNA 1	1782	NC_004018
		Gremmeniella abietina RNA virus MS1 RNA 2	1586	KJ786411
		Gremmeniella abietina RNA virus MS1 RNA 2	1586	NC_004019
		Gremmeniella abietina RNA virus MS1 RNA 3	1187	KJ786412
		Gremmeniella abietina RNA virus MS1 RNA 3	1186	NC_004020
		Gremmeniella abietina RNA virus MS2 unnamed segment 1	1781	AY615211
		Gremmeniella abietina RNA virus MS2 unnamed segment 2	1586	AY615212
		Gremmeniella abietina RNA virus MS2 unnamed segment 3	1186	AY615213
		Penicillium stoloniferum virus F segment 1	1677	NC_007221
		Penicillium stoloniferum virus F segment 2	1500	NC_007222
		Penicillium stoloniferum virus S segment 1	1754	NC_005976
		Penicillium stoloniferum virus S segment 2	1582	NC_005977
	unclassified	Alternaria alternata partitivirus 1 RNA 1	1833	KY352402
		Alternaria alternata partitivirus 1 RNA 2	1680	KY352403
		Amasya cherry disease-associated mycovirus CP	1839	NC_006440
		Amasya cherry disease-associated mycovirus RdRp	2002	NC_006441
		Arabidopsis halleri partitivirus 1 segment 1	1959	NC_030889
		Arabidopsis halleri partitivirus 1 segment 2	1763	NC_030890
		Aspergillus fumigatus partitivirus 1 CP	1623	FN398100

virus family	virus genus	virus species	genomic (segment) RNA length (bp)	Accession number
		Aspergillus fumigatus partitivirus 1 RdRp	1779	FN376847
		Bipolaris maydis partitivirus 1 RNA 1	1930	NC_034514
		Bipolaris maydis partitivirus 1 RNA 2	1790	NC_034524
		Botryosphaeria dothidea partitivirus 1 CP	1623	KF688741
		Botryosphaeria dothidea partitivirus 1 RdRp	1823	KF688740
		Botryosphaeria dothidea virus 1 RNA 1	1757	KJ722537
		Botryosphaeria dothidea virus 1 RNA 2	1392	KJ722538
		Botryosphaeria dothidea virus 1 RNA 3	1124	KJ722539
		Botryotinia fuckeliana partitivirus 1 segment 1	1793	NC_010349
		Botryotinia fuckeliana partitivirus 1 segment 2	1566	NC_010350
		Botryotinia fuckeliana partitivirus 1 segment 3	1383	NC_010351
		Botrytis cinerea partitivirus 1 CP	2219	KF201714
		Carnation cryptic virus 3 RNA 1	1573	NC_034513
		Carnation cryptic virus 3 RNA 2	1562	NC_034523
		Ceratocystis polonica partitivirus CP	2252	AY260757
		Ceratocystis polonica partitivirus RdRp	2156	AY260756
		Ceratocystis polonica partitivirus segment 1	2315	NC_010705
		Ceratocystis polonica partitivirus segment 2	2252	NC_010706
		Colletotrichum partitivirus 1 CP	1535	KP900885
		Colletotrichum truncatum partitivirus 1 RdRp	1820	KR074421
		Flammulina velutipes isometric virus RdRp gene	1919	AB428575
		Fusarium poae partitivirus 2 segment 1	2403	NC_030878
		Fusarium poae partitivirus 2 segment 2	2413	NC_030879
		Fusarium solani partitivirus 2 RdRp	1950	LC006130
		Grapevine associated partitivirus-1 RdRp	2231	GU108584
		Grapevine partitivirus RdRp	2319	JX658566
		Grapevine partitivirus RdRp	1945	JX658569
		Grapevine partitivirus RdRp	1775	JX658570
		Grapevine partitivirus RdRp	2261	JX658571
		Heterobasidion partitivirus 12 CP	1806	KF963176
		Heterobasidion partitivirus 12 RdRp	1884	KF963175
		Heterobasidion partitivirus 13 CP	1776	KF963178
		Heterobasidion partitivirus 13 CP	1775	KF963180
		Heterobasidion partitivirus 13 RdRp	1873	KF963177
		Heterobasidion partitivirus 13 RdRp	1872	KF963179
		Heterobasidion partitivirus 14 RdRp	1886	KF963185
		Heterobasidion partitivirus 15 CP	1791	KF963187
		Heterobasidion partitivirus 15 RdRp	1882	KF963186

virus family	virus genus	virus species	genomic (segment) RNA length (bp)	Accession number
		Heterobasidion partitivirus 7 CP	2231	JN606090
		Heterobasidion partitivirus 7 RdRp	2297	JN606091
		Heterobasidion partitivirus 9 RdRp	2030	JN606085
		Heterobasidion RNA virus 4 RdRp	2007	HQ541325
		Heterobasidion RNA virus 5 RdRp	2000	HQ541326
		Partitivirus-like 3 RdRp	1705	KX148577
		Penicillium aurantiogriseum partitivirus 1 RNA 1	1762	NC_028499
		Penicillium aurantiogriseum partitivirus 1 RNA 2	1576	NC_028494
		Pseudogymnoascus destructans partitivirus-pa RNA 1	1761	KY207543
		Pseudogymnoascus destructans partitivirus-pa RNA 2	1590	KY207544
		Pseudogymnoascus destructans virus CP	1524	NC_030479
		Pseudogymnoascus destructans virus RdRp	1682	NC_030480
		Radish partitivirus JC-2004 RdRp	1866	AY748911
		Raphanus sativus partitivirus 1 RdRp	1915	KT285019
		Rhizoctonia fumigata partitivirus CP	1802	KM668043
		Rhizoctonia fumigata partitivirus RdRp	2003	KM668042
		Rhizoctonia solani dsRNA virus 2 segment 1	2020	NC_023684
		Rhizoctonia solani dsRNA virus 2 segment 2	1810	NC_023685
		Rose partitivirus RNA 1	1937	KU896858
		Rose partitivirus RNA 2	1811	KU896859
		Rosellinia necatrix partitivirus 3 CP	2247	LC010951
		Rosellinia necatrix partitivirus 3 RdRp	2315	LC010950
		Rosellinia necatrix partitivirus 3 RNA 1	2254	AB698491
		Rosellinia necatrix partitivirus 4 RNA 1	2418	AB698493
		Rosellinia necatrix partitivirus 5 RNA 1	2028	AB698494
		Rosellinia necatrix partitivirus 7 segment 1	1977	LC076694
		Rosellinia necatrix partitivirus 7 segment 2	1896	LC076695
		Sclerotinia sclerotiorum partitivirus 1 CP	2292	JX297510
		Sclerotinia sclerotiorum partitivirus 1 RdRp	2334	JX297511
		Sclerotinia sclerotiorum partitivirus S segment 1	1874	NC_013014
		Sclerotinia sclerotiorum partitivirus S segment 2	1852	NC_013015
		Sophora japonica powdery mildew-associated partitivirus CP	1918	NC_030876
		Sophora japonica powdery mildew-associated partitivirus RdRp	1955	NC_030875
		Ustilaginoidea virens partitivirus 2 CP	2082	KF680479
		Ustilaginoidea virens partitivirus 2 RdRp	2112	KF680478
		Ustilaginoidea virens partitivirus 2 segment 1	1712	NC_021873
		Ustilaginoidea virens partitivirus 2 segment 2	1352	NC_021874
		Ustilaginoidea virens partitivirus 3	1711	KC469948

virus family	virus genus	virus species	genomic (segment) RNA length (bp)	Accession number
		Ustilaginoidea virens partitivirus CP	1604	KF805087
		Ustilaginoidea virens partitivirus segment 1	1775	KC503898
		Ustilaginoidea virens partitivirus segment 2	1588	KC503899
		Ustilaginoidea virens partitivirus segment 3	1352	KC503900
		Ustilaginoidea virens partitivirus segment 4	1119	KC503901
		Verticillium albo-atrum partitivirus-1 RNA 1	1730	KJ476945
		Verticillium albo-atrum partitivirus-1 RNA 2	1517	KJ476946
		Verticillium dahliae partitivirus 1 CP	1587	KC422243
		Verticillium dahliae partitivirus 1 RdRp	1768	KC422244
		Verticillium dahliae partitivirus 1 RNA 1	1767	NC_027822
		Verticillium dahliae partitivirus 1 RNA 2	1574	NC_027823
		Vicia faba partitivirus 1 RNA 3	1915	DQ910762
Reoviridae	Mycoreovirus	Cryphonectria parasitica mycoreovirus 2 segment 3	3213	DQ902580
		Mycoreovirus 1 segment 1	4127	NC_010743
		Mycoreovirus 1 segment 10	975	NC_010752
		Mycoreovirus 1 segment 11	732	NC_010753
		Mycoreovirus 1 segment 2	3846	NC_010744
		Mycoreovirus 1 segment 3	3258	NC_010745
		Mycoreovirus 1 segment 4	2269	NC_010746
		Mycoreovirus 1 segment 5	2056	NC_010747
		Mycoreovirus 1 segment 6	2023	NC_010748
		Mycoreovirus 1 segment 7	1539	NC_010749
		Mycoreovirus 1 segment 8	1536	NC_010750
		Mycoreovirus 1 segment 9	1072	NC_010751
		Mycoreovirus 3 segment 10	1171	NC_007529
		Mycoreovirus 3 segment 11	1003	NC_007531
		Mycoreovirus 3 segment 12	943	NC_007532
		Mycoreovirus 3 segment 6	2030	NC_007525
		Mycoreovirus 3 segment 7	1509	NC_007526
		Mycoreovirus 3 segment 8	1299	NC_007527
		Mycoreovirus 3 segment 9	1226	NC_007528
		Mycoreovirus 3 segment S1	4143	NC_007535
		Mycoreovirus 3 segment S2	3773	NC_007533
		Mycoreovirus 3 segment S3	3310	NC_007536
		Mycoreovirus 3 segment S5	2089	NC_007534
		Mycoreovirus 3 segment X	2259	NC_007524
		Sclerotinia sclerotiorum mycoreovirus 4 segment 1	4143	NC_030158
		Sclerotinia sclerotiorum mycoreovirus 4 segment 10	1170	NC_030155

virus family	virus genus	virus species	genomic (segment) RNA length (bp)	Accession number
		Sclerotinia sclerotiorum mycoreovirus 4 segment 11	1093	NC_030156
		Sclerotinia sclerotiorum mycoreovirus 4 segment 12	1000	NC_030157
		Sclerotinia sclerotiorum mycoreovirus 4 segment 2	3775	NC_030161
		Sclerotinia sclerotiorum mycoreovirus 4 segment 3	3307	NC_030162
		Sclerotinia sclerotiorum mycoreovirus 4 segment 4	2259	NC_030159
		Sclerotinia sclerotiorum mycoreovirus 4 segment 5	2084	NC_030163
		Sclerotinia sclerotiorum mycoreovirus 4 segment 6	2029	NC_030160
		Sclerotinia sclerotiorum mycoreovirus 4 segment 7	1512	NC_030152
		Sclerotinia sclerotiorum mycoreovirus 4 segment 8	1304	NC_030153
		Sclerotinia sclerotiorum mycoreovirus 4 segment 9	1223	NC_030154
Totiviridae	Leishmaniavirus	Leishmania aethiopica RNA virus	5193	NC_024115
		Leishmania RNA virus 1	2480	JX313126
		Leishmania RNA virus 1	3228	JX313127
		Leishmania RNA virus 1	4969	KC862308
		Leishmania RNA virus 1 - 1	5284	NC_002063
		Leishmania RNA virus 1 - 4	5283	NC_003601
		Leishmania RNA virus 2	5048	KF256264
		Leishmania RNA virus 2	5048	KF256265
		Leishmania RNA virus 2 - 1	5241	NC_002064
	Totivirus	Anopheles totivirus	6364	KX148550
		Black raspberry virus F	5077	NC_009890
		Botryotinia fuckeliana totivirus 1	5261	NC_009224
		Culex tritaeniorhynchus totivirus	7612	KX456218
		Culex tritaeniorhynchus totivirus	7624	KX456219
		Delisea pulchra totivirus IndA	2463	KT455449
		Delisea pulchra totivirus IndA	2334	KT455450
		Delisea pulchra totivirus IndA	2460	KT455451
		Drosophila melanogaster totivirus SW-2009a	6780	NC_013499
		Golden shiner totivirus	7788	NC_030295
		Grapevine associated totivirus-1	4218	GU108585
		Grapevine associated totivirus-2	6595	GU108594
		Grapevine associated totivirus-2	4967	GU108595
		Gremmeniella abietina RNA virus L2	5129	NC_005965
		Maize-associated totivirus 1	3956	NC_028044
		Maize-associated totivirus 2	4713	NC_030398
		Penicillium aurantiogriseum totivirus 1	5181	NC_028948
		Phomopsis longicolla totivirus 1	4005	KP900901
		Red clover powdery mildew-associated totivirus 1	4673	LC075486

virus family	virus genus	virus species	genomic (segment) RNA length (bp)	Accession number
		Red clover powdery mildew-associated totivirus 1	4781	NC_028480
		Red clover powdery mildew-associated totivirus 2	4178	NC_028481
		Red clover powdery mildew-associated totivirus 3	4784	NC_028483
		Red clover powdery mildew-associated totivirus 4	4455	LC075489
		Red clover powdery mildew-associated totivirus 5	5056	NC_028485
		Red clover powdery mildew-associated totivirus 6	5007	NC_028486
		Red clover powdery mildew-associated totivirus 7	4942	NC_028488
		Red clover powdery mildew-associated totivirus 8	5065	LC075493
		Red clover powdery mildew-associated totivirus 9	5997	NC_028490
		Saccharomyces cerevisiae virus L-A	4580	M28353
		Saccharomyces cerevisiae virus L-A	4579	NC_003745
		Saccharomyces cerevisiae virus L-A CP	540	EF025209
		Saccharomyces cerevisiae virus L-A CP	819	M24336
		Saccharomyces cerevisiae virus L-A-2	4580	KC677754
		Saccharomyces cerevisiae virus L-A-28	4584	KU845301
		Saccharomyces cerevisiae virus L-A-lus	4580	JN819511
		Saccharomyces cerevisiae virus L-BC	4615	NC_001641
		Saccharomyces cerevisiae virus L-BC-2	4614	KX906605
		Saccharomyces cerevisiae virus L-BC-lus	4614	KT784813
		Scheffersomyces segobiensis virus L	4590	KC610514
		Tianjin totivirus	7611	NC_017084
		Tuber aestivum virus 1	4587	HQ158596
		Ustilago maydis virus	1034	L76358
		Ustilago maydis virus H1	6099	NC_003823
		Ustilago maydis virus P1	1504	M63149
		Ustilago maydis virus P4	1004	L12226
		Ustilago maydis virus P4 KP4	576	U25179
		Ustilago maydis virus P6	1234	M27418
		Xanthophyllomyces dendrorhous virus L1A	4655	NC_020903
		Xanthophyllomyces dendrorhous virus L1b	4619	JN997473
		Xanthophyllomyces dendrorhous virus L2	3666	JN997474
	unclassified	Armigeres subalbatus virus SaX06-AK20	7510	NC_014609
		Camponotus nipponicus virus	5713	NC_029312
		Diatom colony associated dsRNA virus 10	5082	AP014902
		Diatom colony associated dsRNA virus 11	5160	AP014903
		Diatom colony associated dsRNA virus 13	4671	AP014905
		Diatom colony associated dsRNA virus 3	4911	AP014893
		Diatom colony associated dsRNA virus 4	4979	AP014895

virus family	virus genus	virus species	genomic (segment) RNA length (bp)	Accession number
		Diatom colony associated dsRNA virus 5	5252	AP014896
		Diatom colony associated dsRNA virus 6	4939	AP014897
		Diatom colony associated dsRNA virus 7	5327	AP014898
		Diatom colony associated dsRNA virus 8	4660	AP014899
		Diatom colony associated dsRNA virus 9 type A	4844	AP014900
		Diatom colony associated dsRNA virus 9 type B	4845	AP014901
		Diatom colony associated virus-Like RNA Segment 3	3458	AP014916
		Diatom colony associated virus-Like RNA Segment 5	3262	AP014918
		Eimeria brunetti RNA virus 1	5358	NC_002701
		Panax notoginseng virus A	5003	NC_029096
		Saccharomyces kudriavzevii virus L-A1	4580	NC_032106
	Victorivirus	Alternaria arborescens victorivirus 1	5206	LC086813
		Aspergillus foetidus slow virus 1	5194	HE588147
		Beauveria bassiana victorivirus 1	5228	HE572591
		Beauveria bassiana victorivirus 1	5329	KR011117
		Beauveria bassiana victorivirus NZL/1980	5327	NC_024151
		Botryosphaeria dothidea victorivirus 1	5322	NC_025214
		Chalara elegans RNA Virus 1	5310	NC_005883
		Coniothyrium minitans mycovirus CP	2389	AF521012
		Coniothyrium minitans RNA virus	4975	NC_007523
		Eimeria tenella RNA virus 1	6006	NC_026140
		Fusarium poae victorivirus 1	5124	NC_030867
		Gremmeniella abietina RNA virus L1	5133	NC_003876
		Helicobasidium mompa totivirus 1-17 segment L	5207	NC_005074
		Helicobasidium mompa totivirus 1-17 segment S	2096	NC_005075
		Helminthosporium victoriae virus 190S	5179	NC_003607
		Magnaporthe oryzae virus 1	5359	NC_006367
		Magnaporthe oryzae virus 2	5193	NC_010246
		Magnaporthe oryzae virus 3	5181	NC_027209
		Nigrospora oryzae victorivirus 1	5100	NC_030224
		Phomopsis vexans RNA virus	5076	NC_026135
		Rosellinia necatrix victorivirus 1	5329	NC_021565
		Sclerotinia nivalis victorivirus 1	5162	NC_030392
		Sphaeropsis sapinea RNA virus 1	5163	NC_001963
		Sphaeropsis sapinea RNA virus 2	5202	NC_001964
		Tolypocladium cylindrosporum virus 1	5196	NC_014823
		Ustilaginoidea virens RNA virus 1	5567	JX524563
		Ustilaginoidea virens RNA virus 1	5220	KF791041

virus family	virus genus	virus species	genomic (segment) RNA length (bp)	Accession number
		Ustilaginoidea virens RNA virus 1	5142	NC_020997
		Ustilaginoidea virens RNA virus 3	5075	NC_023547
		Ustilaginoidea virens RNA virus 5	5221	NC_028477
		Ustilaginoidea virens RNA virus L	5654	NC_025366
		Ustilaginoidea virens RNA virus M	2714	NC_025367
unclassified	Alternavirus	Fusarium poae alternavirus 1 segment 1	3559	NC_030883
		Fusarium poae alternavirus 1 segment 2	2496	NC_030880
		Fusarium poae alternavirus 1 segment 3	2482	NC_030881
	Botybirnavirus	Sclerotinia sclerotiorum botybirnavirus 2 RNA 1	6159	KT962972
		Sclerotinia sclerotiorum botybirnavirus 2 RNA 2	5872	KT962973
	Fusarivirus	Fusarium poae fusarivirus 1	6379	NC_030868
	Megabirnavirus	Rosellinia necatrix megabirnavirus 2-W8 segment 1	8985	NC_029125
		Rosellinia necatrix megabirnavirus 2-W8 segment 2	7959	NC_029126
		Sclerotinia sclerotiorum megabirnavirus 1	8806	NC_027221
		Sclerotinia sclerotiorum megabirnavirus 1	7909	NC_027222
	Tetramycovirus	Aspergillus fumigatus tetramycovirus-1 RNA 1	2403	HG975302
		Aspergillus fumigatus tetramycovirus-1 RNA 2	2233	HG975303
		Aspergillus fumigatus tetramycovirus-1 RNA 3	1970	HG975304
		Aspergillus fumigatus tetramycovirus-1 RNA 4	1131	HG975305
	unclassified	Cladosporium cladosporioides virus 1 RNA 1	2434	NC_024704
		Cladosporium cladosporioides virus 1 RNA 2	2241	NC_024705
		Cladosporium cladosporioides virus 1 RNA 3	2008	NC_024706
		Cladosporium cladosporioides virus 1 RNA 4	1261	NC_024707
		Cladosporium cladosporioides virus 1 RNA 5	942	NC_024708
		Fusarium graminearum dsRNA mycovirus-1	6621	NC_006937
		Fusarium poae dsRNA virus 2	9518	NC_030201
		Fusarium poae dsRNA virus 3	9419	NC_030202
		Fusarium poae mycovirus 1	9148	NC_030869
		Fusarium poae mycovirus 2	4224	NC_030870
		Nigrospora oryzae unassigned RNA virus 1	2857	KT258976
		Saccharomyces cerevisiae killer virus M1	1801	NC_001782
		Saccharomyces cerevisiae killer virus Mlus	2033	GU723494
